# Therapeutic efficacy and biodistribution of allogeneic mesenchymal stem cells delivered by intrasplenic and intrapancreatic routes in streptozotocin-induced diabetic mice

**DOI:** 10.1186/s13287-015-0017-1

**Published:** 2015-03-14

**Authors:** Juliana Navarro Ueda Yaochite, Carolina Caliari-Oliveira, Lucas Eduardo Botelho de Souza, Lourenço Sbragia Neto, Patrícia Vianna Bonini Palma, Dimas Tadeu Covas, Kelen Cristina Ribeiro Malmegrim, Julio César Voltarelli, Eduardo Antônio Donadi

**Affiliations:** Department of Biochemistry and Immunology, Basic and Applied Immunology Program, School of Medicine of Ribeirão Preto, University of São Paulo, Av. Bandeirantes 3900, Monte Alegre 14049-900, Ribeirão Preto, São Paulo Brazil; Tenente Catão Roxo 2501, Monte Alegre 14051-140, Ribeirão Preto, São Paulo Brazil; Department of Clinical Medicine, School of Medicine of Ribeirão Preto, University of São Paulo, Av. Bandeirantes 3900, Monte Alegre 14049-900, Ribeirão Preto, São Paulo Brazil; Department of Surgery and Anatomy, Pediatric Surgery Division, School of Medicine of Ribeirão Preto, University of São Paulo, Av. Bandeirantes 3900, Monte Alegre 14049-900, Ribeirão Preto, São Paulo Brazil; Regional Blood Center of Ribeirão Preto, University of São Paulo, Tenente Catão Roxo 2501, Monte Alegre 14051-140, Ribeirão Preto, São Paulo Brazil; Department of Clinical, Toxicological and Bromatological Analysis, School of Pharmaceutical Sciences of Ribeirão Preto, University of São Paulo, Av. do Café, Monte Alegre 14040-903, Ribeirão Preto, São Paulo Brazil

## Abstract

**Introduction:**

Mesenchymal stromal/stem cells (MSCs) are multipotent cells that have the ability to express and secrete a wide range of immunomodulatory molecules, cytokines, growth factors and antiapoptotic proteins. MSCs modulate both innate and adaptive immune responses making them potential candidates for the treatment of patients with type 1 diabetes mellitus (T1D). However, one problem frequently associated with the systemic MSCs administration is the entrapment of the cells mainly in the lungs. In this sense, trying to avoid the lung barrier, the purpose of this study was to evaluate the long-term therapeutic efficacy and biodistribution of allogeneic adipose tissue-derived MSCs (ADMSCs) injected via two different delivery routes (intrasplenic/I.Sp and intrapancreatic/I.Pc) in a murine model of diabetes induced by streptozotocin (STZ).

**Methods:**

Experimental diabetes was induced in C57BL/6 male mice by multiple low-doses of STZ. MSCs were isolated from adipose tissue (ADMSCs) of Balb/c mice. A single dose of 1x10^6^ ADMSCs was microinjected into the spleen or into the pancreas of diabetic mice. Control group received injection of PBS by I.Sp or I.Pc delivery routes. Glycemia, peripheral glucose response, insulin-producing β cell mass, regulatory T cell population, cytokine profile and cell biodistribution were evaluated after ADMSCs/PBS administration.

**Results:**

ADMSCs injected by both delivery routes were able to decrease blood glucose levels and improve glucose tolerance in diabetic mice. ADMSCs injected by I.Sp route reverted hyperglycemia in 70% of diabetic treated mice, stimulating insulin production by pancreatic β cells. Using the I.Pc delivery route, 42% of ADMSCs-treated mice responded to the therapy. Regulatory T cell population remained unchanged after ADMSCs administration but pancreatic TGF-β levels were increased in ADMSCs/I.Sp-treated mice. ADMSCs administrated by I.Sp route were retained in the spleen and in the liver and ADMSCs injected by I.Pc route remained in the pancreas. However, ADMSCs injected by these delivery routes remained only few days in the recipients.

**Conclusion:**

Considering the potential role of MSCs in the treatment of several disorders, this study reports alternative delivery routes that circumvent cell entrapment into the lungs promoting beneficial therapeutic responses in ADMSCs-treated diabetic mice.

**Electronic supplementary material:**

The online version of this article (doi:10.1186/s13287-015-0017-1) contains supplementary material, which is available to authorized users.

## Introduction

Stem cell-based therapies, which involve replacement, repair or enhancement of the biological function of a damaged organ or tissue, have emerged as a potent therapeutic strategy for many diseases [1]. These therapies may represent an alternative approach to insulin, pancreas and pancreatic islet transplantations in the treatment of patients with type 1 diabetes mellitus (T1D), and adult stem cells (such as hematopoietic and mesenchymal stem cells) represent an attractive and promising tool for this purpose [2,3].

Mesenchymal stromal/stem cells (MSCs) are multipotent cells that have the ability to differentiate into cells from mesodermal lineage such as adipocytes, chondroblasts and osteoblasts [4], and they can be isolated and expanded with high efficiency from several adult and fetal tissues, including bone marrow, adipose tissue, dental pulp and umbilical cord blood [4,5]. Adipose tissue-derived mesenchymal stem cells (ADMSCs) are obtained in larger quantities than MSCs isolated from other tissues [6]. They can easily be expanded *in vitro* and exhibit regenerative properties after injection into experimental models of autoimmune encephalomyelitis, collagen-induced arthritis, colitis, spontaneous diabetes and others diseases [7-10].

MSCs have been shown to express and secrete a wide range of immunomodulatory molecules, cytokines, growth factors and antiapoptotic proteins. These molecules play vital roles in MSC paracrine function and contribute to tissue repair and homeostasis through mechanisms involving cytoprotection, immunomodulation, neovascularization and inhibition of apoptosis [11-13]. Regarding the immunomodulatory properties of MSCs, the ability to modulate both innate and adaptive immune responses makes them potential candidates for the treatment of patients with T1D.

MSCs have been widely tested in spontaneous and chemically-induced experimental models of T1D. The administration of MSCs promoted hyperglycemia reversion, pancreatic islet repair, insulin production improvement, regulatory T (Treg) cell expansion and inflammatory process reduction in MSC-treated diabetic animals [7,14-21]. Most of these studies injected MSCs using the intravenous route of administration. However, one problem frequently associated with the systemic delivery routes (intravenous and intra-arterial) is the entrapment of the cells mainly in the lungs [22,23]. Systemically injected MSCs are trapped within the pulmonary capillaries, causing pulmonary and hemodynamic alterations, and preventing the intended access to other organs [24]. This phenomenon is due to the mean size of injected MSCs being larger than the diameter of pulmonary capillaries [24,25], and also seems to be related to the interactions of MSC adhesion molecules with their ligands in the endothelium [26,27].

This initial pulmonary entrapment might alter the migratory ability of the cells leading to nonspecific accumulation, especially in the reticuloendothelial system [26]. To enhance therapeutic success, while avoiding microembolization, future efforts should explore alternative approaches that preserve the ability of MSCs to migrate, survive and efficiently achieve the target organ [25]. In this sense, trying to avoid the lung barrier, the purpose of this study was to evaluate long-term therapeutic efficacy and the biodistribution of allogeneic ADMSCs injected via two different routes of administration – intrasplenic (i.sp.) and intrapancreatic (i.pc.) – in a murine model of streptozotocin (STZ)-induced diabetes.

## Methods

### Experimental design

Experiments were designed according to the protocol represented in Additional file 1.

### Animals

C57BL/6, Balb/c and FVB-Tg (CAG-luc,-GFP)L2G85Chco/J (FVB^Luc+^) mice were purchased from The Jackson Laboratory (Bar Harbor, ME, USA) and housed at constant temperature and humidity, with a 12 hours:12 hours light–dark cycle, and food and water were available *ad libitum*. All protocols were conducted in accordance with the Brazilian Committee for Experimental Animals and were approved by the Ethics Committee for Animal Research of the School of Medicine of Ribeirão Preto, at the University of São Paulo (# 157/2010, # 021/2013-01).

### Isolation of adipose tissue-derived MSCs and ADMSCs^Luc+^

ADMSCs were isolated from inguinal and epididymal fat tissues from 8-week-old to 10-week-old male Balb/c and FVB^Luc+^ mice. First, fat tissue samples were intensely washed with phosphate-buffered saline (PBS). The tissues were then cut into small pieces and digested with 1 mg/ml type 1A collagenase (Sigma-Aldrich, St. Louis, MO, USA) for 60 minutes at 37°C. The collagenase activity was neutralized with Dulbecco’s Modified Eagle’s medium (Gibco Life Technologies, Grand Island, NY, USA) containing 15% fetal bovine serum (FBS; Thermo Scientific, Rockford, IL, USA). The digested adipose tissue was centrifuged and the pellet was resuspended in PBS and filtered through a 100 μm nylon cell strainer (BD, Franklin Lakes, NJ, USA). The filtered cells were centrifuged, resuspended and cultured with Dulbecco’s Modified Eagle’s medium low-glucose medium (Gibco) supplemented with 15% FBS, 100 μg/ml penicillin (Gibco), 100 μg/ml streptomycin (Gibco) and 2 mM l‐glutamine (Gibco). Nonadherent cells were removed 3 days after initial plating by replacing the medium, and the ADMSCs were subcultured until the fourth passage.

### Characterization of adipose tissue-derived MSCs and ADMSCs^Luc+^

The morphology, immunophenotypic profile and *in vitro* differentiation potential (adipocyte and osteocytes) of ADMSCs/ADMSCs^Luc+^ were characterized at the fourth passage.

ADMSCs/ADMSCs^Luc+^ were incubated with anti-mouse CD29, CD90.2, CD105, CD73, CD34, CD45, CD11b, CD117, PDGF and CD31monoclonal antibodies (BD) for 30 minutes at room temperature. Cells were then analyzed by a FACSCalibur™ cytometer (BD) using CellQuest Pro software (BD).

To induce adipogenic differentiation, confluent adherent ADMSCs/ADMSCs^Luc+^ were cultured in α-Minimum Essential Medium (Gibco), supplemented with 15% FBS, 100 mM dexamethasone (Prodome, Campinas, SP, Brazil), 10 μg/ml insulin (Sigma‐Aldrich) and 100 μM indomethacin (Sigma‐Aldrich), and replaced every 3 days. After 15 days of differentiation induction, cells were then fixed and stained with Sudan II-Scarlet and Harris hematoxylin.

To induce osteogenic differentiation, confluent adherent ADMSCs/ADMSCs^Luc+^ were cultured in α-Minimum Essential Medium (Gibco), and supplemented with 7.5% FBS, 1 μM dexamethasone (Prodome), 200 μM ascorbic acid 2-phosphate (Sigma-Aldrich) and 10 mM β-glycerophosphate (Sigma-Aldrich). After 21 days of stimulation, cell differentiation was confirmed by von Kossa staining.

### Experimental diabetes model

C57BL/6 male mice at 10 weeks of age were intraperitoneally injected with 40 mg/kg STZ (Sigma-Aldrich) for 5 consecutive days. Blood samples were taken from the tail vein and glucose levels were frequently monitored with an Accu-Chek Active glucometer (Roche, Roche Diagnostics, Abbott Park, IL, USA) under nonfasting conditions. Mice were considered to be diabetic when nonfasting blood glucose levels were higher than 250 mg/dl for two sequential determinations.

### Intrasplenic injection of adipose tissue-derived MSCs

For i.sp. injection of MSCs, mice were anesthetized with a mixture of ketamine (Ketamina-Agener União, São Paulo, Brazil) and xylazine (Dopaser-Hertape Calier, Minas Gerais, Brazil). Incisions in the skin and peritoneum were made and the spleen was totally exposed. A single dose of 1 × 10^6^ ADMSCs suspended in 70 μl PBS + Pulmozyme (Dornase alpha-rhDNase; Roche) was microinjected into the spleen of diabetic mice (ADMSCs/i.sp., *n* = 10) 20 days after the last dose of STZ. DNase (Pulmozyme) was used to promote the degradation of DNA released by disrupted cells avoiding MSC aggregation. At the same time, a control group of diabetic mice was injected with PBS + Pulmozyme (Control-PBS, *n* = 5) by the i.sp. route. Bleeding was controlled using a cotton swab and local application of fibrin sealant. The incisions were sutured using a 5–0 nylon monofilament (Bioline Fios Cirúrgicos Ltda, Goiás, Brazil). Intraperitoneal administration of 30 mg/kg tramadol hydrochloride (Tramal, Medley, Campinas, Brazil) was used as a pain reliever every 12 hours for 3 consecutive days. Mice were sacrificed 70 days after i.sp. ADMSC/PBS administration.

### Intrapancreatic injection of adipose tissue-derived MSCs

For i.pc. injection of ADMSCs, mice were anesthetized as described above. Incisions in the skin and peritoneum were made and the pancreas was totally exposed. A total of 1 × 10^6^ ADMSCs in 100 μl PBS + Pulmozyme (Roche) was injected in different points along the pancreas of diabetic mice (ADMSCs/i.pc., *n* = 12) 20 days after the last dose of STZ. At the same time, a control group of diabetic mice was injected with PBS + Pulmozyme (Control-PBS, *n* = 5) by the i.pc. route. The incisions were sutured and tramadol hydrochloride was used as a pain reliever. Mice were sacrificed 70 days after i.pc. ADMSC/PBS administration.

### Intraperitoneal glucose tolerance test

The peripheral response to glucose was evaluated by glucose tolerance tests (GTT) performed 65 days after ADMSC transplantation. A solution of glucose (1.5 mg/g body weight) was intraperitoneally administrated in mice fasting for 10 hours, and blood glucose levels were determined before and 15, 30, 60, 120 and 180 minutes after glucose administration.

### Histology and immunohistochemistry analysis

For histologic analysis, paraffin-embedded pancreatic sections were stained with hematoxylin and eosin. Immunohistochemistry reactions were performed on paraffin-embedded sections. First, the sections were incubated with Peroxidase-Blocking Reagent (DAKO Cytomation, Fort Collins, CO, USA) followed by incubation with PBS + 1% bovine serum albumin (Sigma-Aldrich) and Triton X-100 (Sigma-Aldrich) to prevent unspecific staining. Next, rabbit monoclonal anti-mouse insulin antibody (Santa Cruz Biotechnology, Santa Cruz, CA, USA) was applied to the sections, followed by incubation with the LSAB™ + Kit/HRP (DAKO Cytomation). The slides were stained with diaminobenzidine according to the manufacturer’s instructions (DAKO Cytomation). Finally, the sections were counterstained with Harris hematoxylin and analyzed by light microscopy.

### Pancreatic islet morphometry

For each animal, 10 pancreatic sections stained with hematoxylin and eosin were randomly chosen and evaluated. The number of islets per section was counted and the whole islet area was determined. All analyses were performed using a computerized system for morphometry (ImageJ; National Institutes of Health, Bethesda, Maryland, USA).

### Analysis of regulatory T-cell population in spleen and pancreatic lymph nodes

First, cells from spleen and pancreatic lymph nodes (PLN) were isolated through mechanical dissociation. Fluorochrome-conjugated primary antibodies for CD4, CD25 and their control isotypes (BD) were added to cell suspensions and incubated for 30 minutes in the dark at room temperature. After extracellular antigen staining, cells were incubated with FACS Lysing solution (BD) for 10 minutes in the dark. Samples were then washed and resuspended in FACS Permeabilizing solution (BD) for 10 minutes. Next, the expression of the transcription factor Foxp3 was assessed by incubating the cells with phycoerythrin-conjugated anti-mouse Foxp3 monoclonal antibody (BD). Cells were analyzed using a FACSCalibur™ flow cytometer (BD) and the frequency of Treg cells (CD4^+^CD25^+^Foxp3^+^) was determined by CellQuest Pro software (BD).

### Quantification of cytokine levels in serum and pancreatic tissue

Pieces of pancreas were removed, weighed and placed into a tube containing Complete Protease Inhibitor Cocktail (Roche Diagnostics, Abbott Park, IL, USA). Pancreatic tissue was homogenized using a Polytron homogenizer (Kinematica, Luzern, Switzerland) and interleukin (IL)-2, IL-6, interferon gamma, IL-17, IL-4 and IL-10 levels were detected in the supernatant by the cytometric bead array method (Th1/Th2/Th17 kit; BD), according to the manufacturer’s instructions. The concentration of transforming growth factor beta (TGF-β) in pancreatic tissue was determined using the Human/Mouse TGF-β1 ELISA Ready-Set-Go kit (eBioscience, San Diego, CA, USA). Serum cytokine levels were also determined by the cytometric bead array method.

### Quantification of circulating-insulin levels

Blood samples of nonfasting mice were collected 70 days after ADMSC/PBS administration. The insulin concentration in the serum was determined using the Mouse Ultrasensitive Insulin ELISA kit (Alpco Diagnostics, Salem, Massachusetts, USA) according to the manufacturer’s instructions.

### Adipose tissue-derived MSC trafficking: bioluminescent imaging

Bioluminescent imaging (BLI) was performed using the Lumina *In Vivo* Imaging System (Perkin Elmer, Waltham, MA, USA). For imaging of cultured ADMSCs^Luc+^, d-luciferin (Perkin Elmer) was added to culture medium to a final concentration of 150 μg/ml prior to imaging. For *in vivo* imaging, diabetic ADMSC/PBS-treated mice were intraperitoneally injected with 150 mg/kg d-luciferin and placed into the *in vivo* imaging system chamber under continuous exposure to 1.5% isoflurane (Abbot Laboratories, São Paulo, SP, Brazil). For *ex vivo* imaging, 150 mg/kg d-luciferin was injected into diabetic treated mice 10 minutes prior necropsy. Next, organs were excised, immersed in a solution of 300 μg/ml d-luciferin and imaged. *In vivo* analysis was performed 0, 1, 3, 5, 8 and 11 days after i.sp. or i.pc. ADMSC/PBS administration and *ex vivo* analysis were performed 2 days after i.sp. or i.pc. ADMSC/PBS injection. For bioluminescence quantification, a region of interest was manually drawn to encompass the bioluminescent signal and the intensity was recorded as photon flux (photons/second).

### Statistical analysis

Data are present as mean ± standard deviation. Statistical comparisons were made by unpaired/paired *t* tests or by one-way analysis of variance with the Tukey post test. *P* <0.05 was considered significant.

## Results

### Characterization of adipose tissue-derived MSCs

ADMSCs isolated from adipose tissue of Balb/c mice exhibited typical spindle fibroblast-like morphology and immunophenotypic profile at the fourth passage. ADMSCs were able to differentiate into adipocytes and osteocytes after culture in specific inductive media, thereby confirming their multipotency (Additional file 2).

### Intrasplenic and intrapancreatic administration of adipose tissue-derived MSCs decrease blood glucose levels in diabetic mice

ADMSCs were administrated by i.sp. or i.pc. injection into mice with established diabetes (20 days after the last dose of STZ). The control group received PBS injection (Control-PBS) by the same routes.

**Figure 1:**
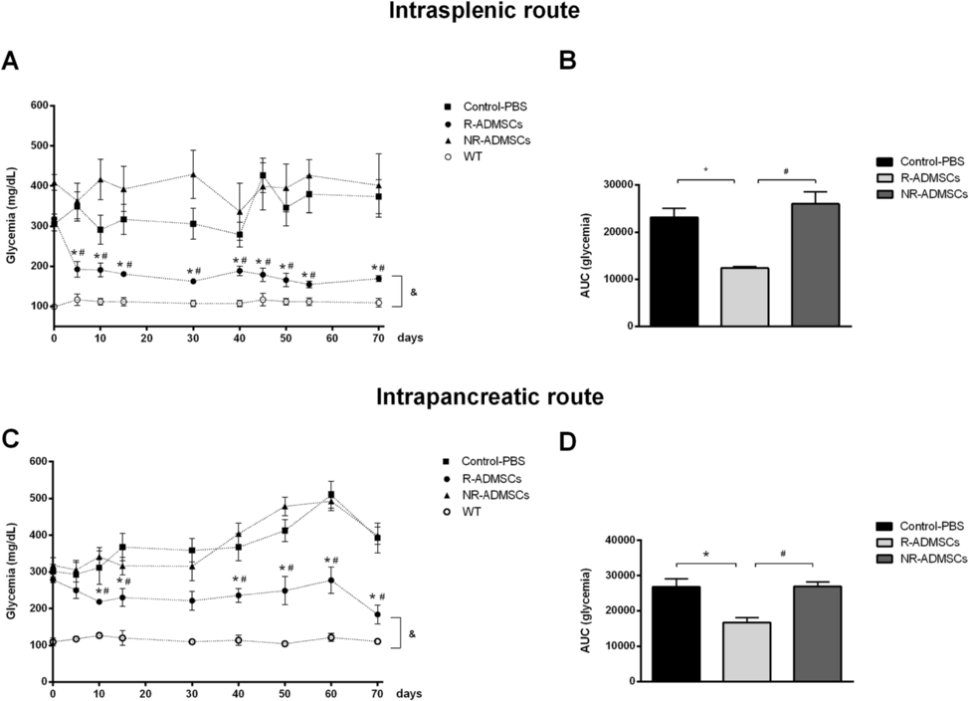
**Intrasplenic and intrapancreatic injections of adipose tissue-derived mesenchymal stem cells promote reversion of hyperglycemia in streptozotocin-induced diabetic mice.** Adipose tissue-derived mesenchymal stem cells (ADMSCs; 1 × 10^6^) were administered by intrasplenic (*n* = 10) or intrapancreatic (*n* = 12) injection in diabetic mice 20 days after diabetes induction. Control groups were treated with injections of phosphate-buffered saline (Control-PBS, *n* = 5). Blood glucose levels (mg/dl) were frequently measured in nonfasting mice. **(A)** Intrasplenic ADMSC administration decreased blood glucose levels in 70% (7/10) of diabetic treated mice (responder: R-ADMSCs). **(C)** Intrapancreatic ADMSC injection reverted diabetes in 42% (5/12) of diabetic treated mice (R-ADMSCs). Nonresponder mice (NR-ADMSCs) remained hyperglycemic throughout the entire experimental period (glycemia >250 mg/dl). WT, wild-type/nondiabetic mice (*n* = 2). **(B, D)** Area under the curve (AUC) of glycemia from day 0 to day 70. The AUC was determined for each animal, and the average ± standard deviation of each group is shown. *P <0.05 (Control-PBS vs. R-ADMSCs), ^#^ *P* <0.05 (R-ADMSCs vs. NR-ADMSCs), &*P* <0.05 (WT vs. R-ADMSCs, for all evaluated time points).

ADMSCs injected by the i.sp. route reversed hyperglycemia in 70% (7/10) of diabetic treated mice, which were identified as responder mice (R-ADMSCs/i.sp.). The remaining three were nonresponder mice (NR-ADMSCs/i.sp.) and exhibited high blood glucose levels throughout the entire experimental period (Figure 1A). The area under the glycemia curve (AUC) of R-ADMSCs/i.sp. mice (12,390 ± 316.3) was significant lower compared with the NR-ADMSCs/i.sp. group (25,960 ± 2,547) and the Control-PBS group (23,150 ± 1,892; *P* = 0.0001; Figure 1B).

The i.pc. injection of ADMSCs was less efficient to reverse experimentally induced diabetes. Following ADMSC administration, 42% (5/12) of diabetic treated mice were responsive to treatment and presented low levels of blood sugar (identified as R-ADMSCs/i.pc.). In contrast, the nonresponder mice (NR-ADMSCs/i.pc.) exhibited high levels of blood glucose similar to the Control-PBS group (Figure 1C). The AUC of R-ADMSCs/i.pc. mice (16,630 ± 1,467) was significant lower compared with the NR-ADMSCs/i.pc. group (26,880 ± 1,298) and the Control-PBS group (26,790 ± 2,253; *P* = 0.0009; Figure 1D).

Despite the improvement in glycemic control promoted by ADMSC therapy, nonfasting blood glucose levels of R-ADMSCs mice (i.sp. and i.pc.) were higher than those presented by nondiabetic mice (wild-type group; Figure 1A,C) during the entire experimental period.

The mean of the body weight of all experimental groups remained unchanged during the follow-up (data not shown).

### Adipose tissue-derived MSC transplantation improves peripheral glucose response in diabetic treated mice

**Figure 2:**
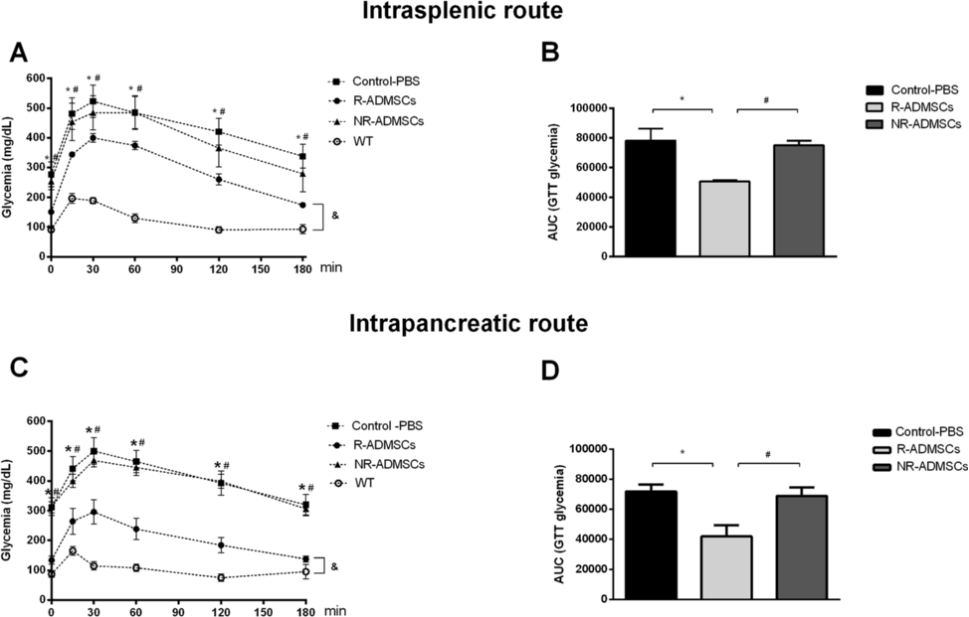
**Intrasplenic and intrapancreatic injections of adipose tissue-derived mesenchymal stem cells improve peripheral response to glucose in streptozotocin-induced diabetic mice.** Glucose tolerance tests (GTTs) were performed in 10-hour fasting mice 65 days after adipose tissue-derived mesenchymal stem cell (ADMSC)/phosphate-buffered saline (PBS) administration. Glucose (1.5 mg/g) was intraperitoneally administered and blood glucose levels (mg/dl) were determined 0, 15, 30, 60, 120 and 180 minutes after administration. **(A, C)**. Responder ADMSC-treated mice (R-ADMSCs) exhibited lower blood glucose levels than control mice (Control-PBS) and nonresponder mice (NR-ADMSCs) during the GTT. WT, wild-type/nondiabetic mice (*n* = 2). **(B, D)** Area under the curve (AUC) of glycemia during the GTT was determined for each animal and the average ± standard deviation of each group is shown. Intrasplenic route: Control-PBS, *n* = 5; R-ADMSCs-treated, *n* = 7; NR-ADMSCs, *n* = 3. Intrapancreatic route: Control-PBS, *n* = 5; R-ADMSCs, *n* = 5; NR-ADMSCs, *n* = 7. *P <0.05 (Control-PBS vs. R-ADMSCs), ^#^ *P* <0.05 (R-ADMSCs vs. NR-ADMSCs), &*P* < 0.05 (WT vs. R-ADMSCs, for all evaluated time points).

The GTTs were performed 65 days after the administration of ADMCs/PBS to evaluate glucose metabolism in diabetic treated mice. The R-ADMSCs/i.sp. mice showed a significant improvement in response to intraperitoneal glucose administration (Figure 2A), and the GTT AUC of the R-ADMSCs/i.sp. group (50,613 ± 2,283) was significantly lower (*P* = 0.01) compared with the Control-PBS AUC (78,260 ± 8,214) and NR-ADMSCs (75,003 ± 5,585) during the test (Figure 2B).

The ADMSCs injected by the i.pc. route also improved the response to glucose in the R-ADMSCs/i.pc. group compared with the NR-ADMSCs/i.pc. and Control-PBS groups. The R-ADMSCs/i.pc. mice exhibited a better GTT curve (Figure 2C) and lower GTT AUC (Figure 2D) than the other experimental groups (*P* = 0.005).

Despite the improvement in peripheral glucose response promoted by ADMSC therapy, the GTT curves of R-ADMSCs mice (i.sp. and i.pc.) were different from the curves of the wild-type group (Figure 2A,C).

### Intrasplenic administration of adipose tissue-derived MSCs improves β-cell mass and insulin production in diabetic treated mice

**Figure 3:**
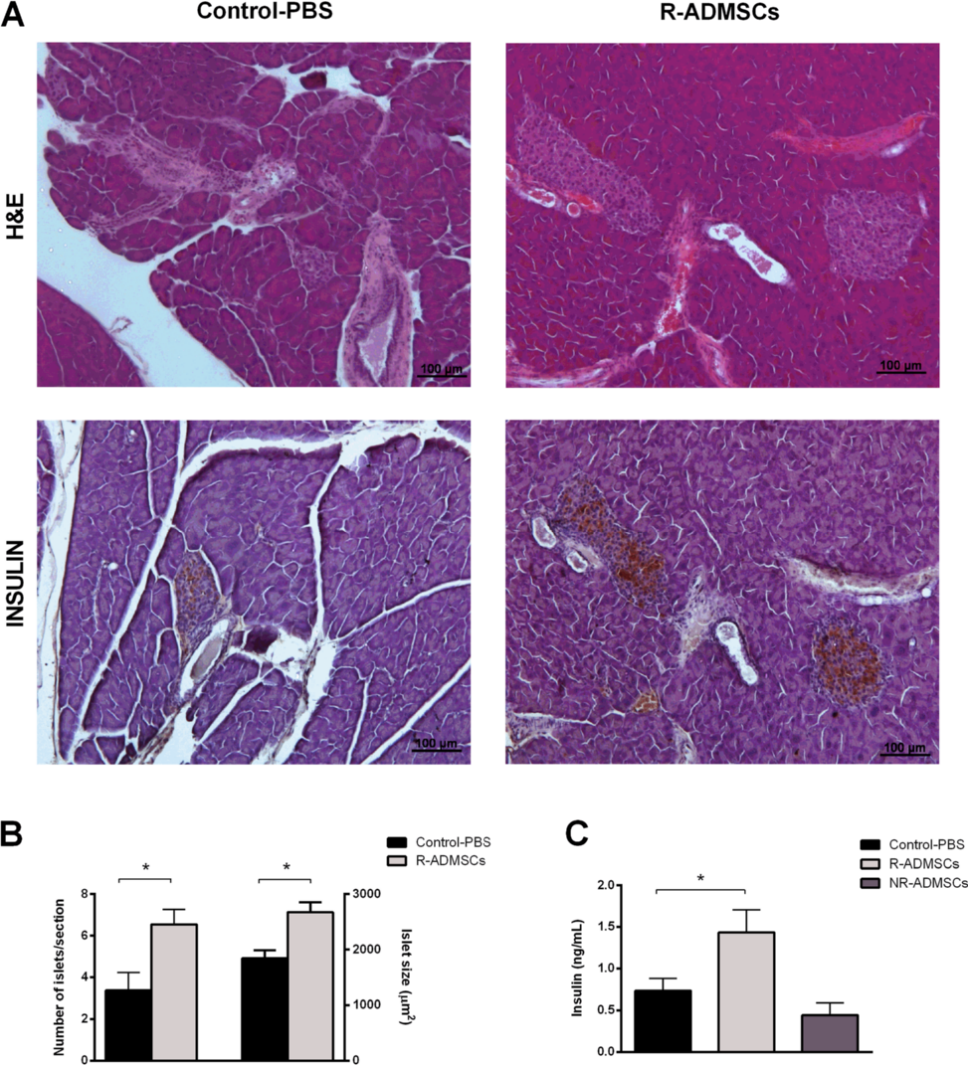
**Intrasplenic adipose tissue-derived mesenchymal stem cell transplantation improves insulin-producing β-cell mass in diabetic treated mice.** Pancreata from Control-PBS and adipose tissue-derived mesenchymal stem cell (ADMSC)-treated mice were collected 70 days after the treatment. Islet morphology was evaluated by hematoxylin and eosin (H&E) staining and the *in situ* insulin content was detected by immunohistochemistry assay. **(A)** Representative H&E and insulin-stained islets from the Control-PBS group (left panel) and the responder ADMSC-treated group (R-ADMSCs; right panel) are shown. Original magnification 100×. **(B)** The number of islets per section (left *y* axis) was counted and the islet area (right *y* axis) was determined. **(C)** Blood samples of nonfasting mice were collected at day 70 post transplantation and circulating-insulin levels were determined by enzyme-linked immunosorbent assay. Bars represent average ± standard deviation. *P <0.05 (Control-PBS vs. R-ADMSCs). NR-ADMSCs, nonresponder ADMSC-treated mice; PBS, phosphate-buffered saline.

Histological analysis of the pancreas 70 days after ADMSC treatment demonstrated that pancreatic islets from R-ADMSC/i.sp. mice exhibited preserved morphology, high levels of *in situ* insulin staining (Figure 3A), and increased islet area and numbers of islets per section (Figure 3B) compared with the pancreatic islets of PBS-treated diabetic mice. In parallel, the serum insulin levels were significantly increased in the R-ADMSCs/i.sp. group (1.43 ± 0.2 ng/ml) compared with the Control-PBS group (0.73 ± 0.1 ng/ml; *P* <0.05; Figure 3C).

**Figure 4:**
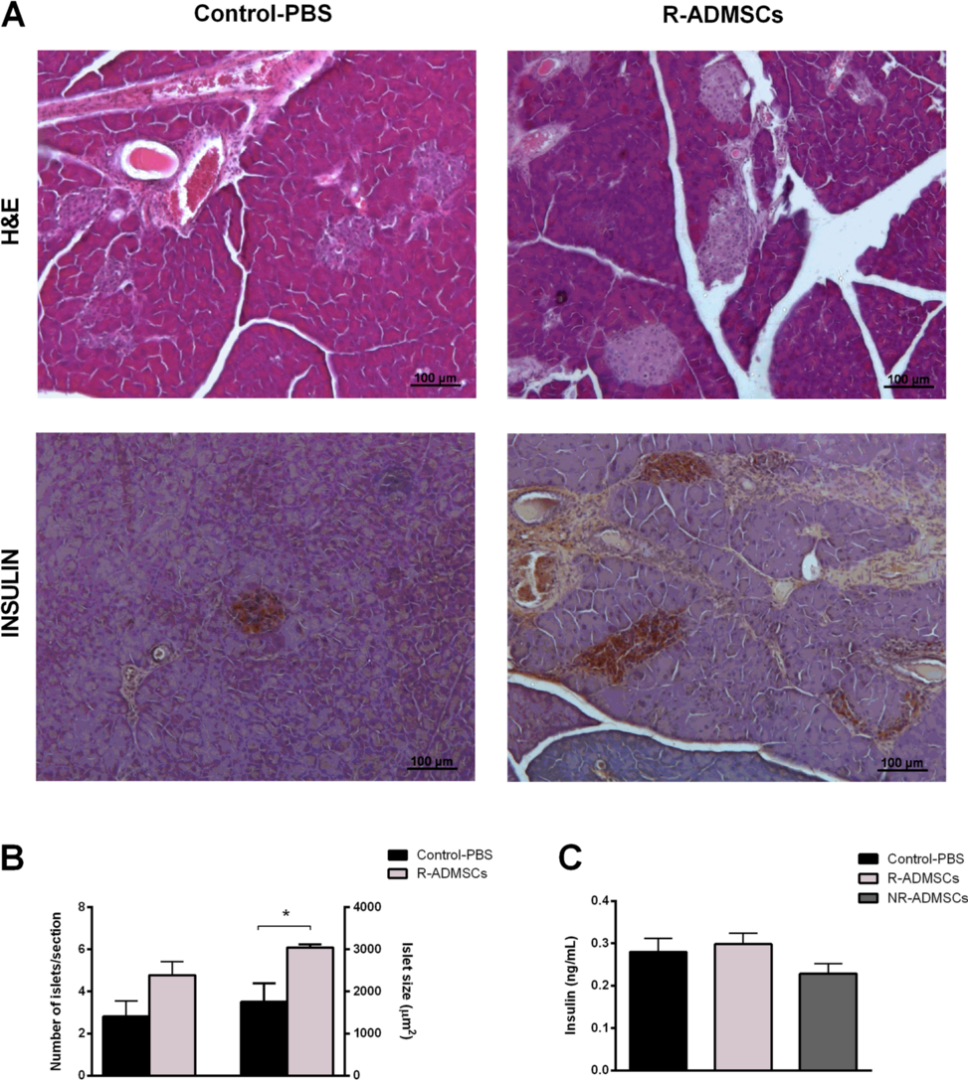
**Intrapancreatic adipose tissue-derived mesenchymal stem cell transplantation does not increase serum insulin levels in diabetic treated mice.** Pancreata from Control-PBS and adipose tissue-derived mesenchymal stem cell (ADMSC)-treated mice were collected 70 days after the treatment. Islet morphology was evaluated by hematoxylin and eosin (H&E) staining and the *in situ* insulin content was detected by immunohistochemistry assay. **(A)** Representative H&E and insulin-stained islets from the Control-PBS group (left panel) and the responder ADMSC-treated group (R-ADMSCs; right panel) are shown. Original magnification 100×. **(B)** The number of islets per section (left *y* axis) was counted and the islet area (right *y* axis) was determined. **(C)** Blood samples of nonfasting mice were collected at day 70 post transplantation and circulating-insulin levels were determined by enzyme-linked immunosorbent assay. Bars represent average ± standard deviation. *P <0.05 (Control-PBS vs. R-ADMSCs). NR-ADMSCs, nonresponder ADMSC-treated mice; PBS, phosphate-buffered saline.

A preserved morphology and increased islet area were also observed in the islets of R-ADMSC/i.pc. mice (Figure 4A,B). However, despite the intense *in situ* insulin expression in pancreatic islets of R-ADMSC/i.pc. mice, the levels of circulating insulin were similar to those observed in the NR-ADMSC/i.pc. and Control-PBS groups (Figure 4C).

The pancreatic islet morphology, morphometry and insulin staining intensity of NR-ADMSCs mice were similar to the Control-PBS groups regardless of the injection strategy (data not shown)*.*

The presence of Ki-67-positive cells, reflecting pancreatic cells under proliferation was similar in all experimental groups 70 days after PBS/ADMSC administration (Additional file 3).

### Adipose tissue-derived MSC administration does not alter the frequency CD4^+^CD25^+^Foxp3^+^ regulatory T cells in diabetic treated mice

**Figure 5:**
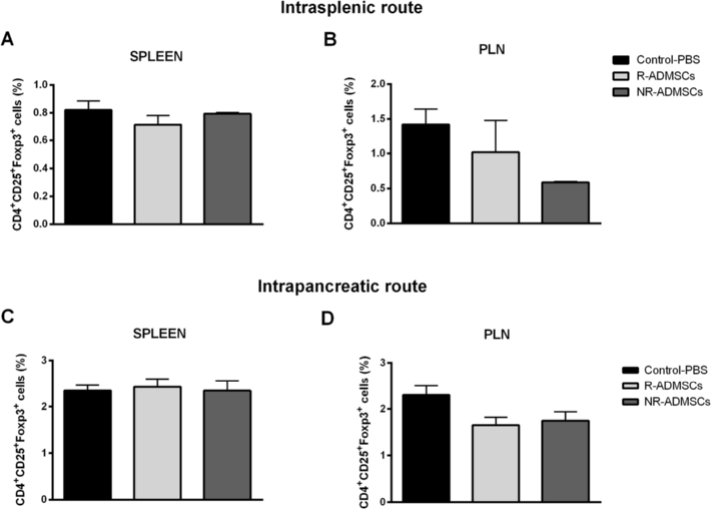
**Intrasplenic and intrapancreatic adipose tissue-derived mesenchymal stem cell administrations do not change the frequency of regulatory T cells in the spleen and pancreatic lymph nodes of diabetic mice 70 days after cell transplantation.** Frequency of regulatory CD4^+^CD25^+^Foxp3^+^ T (Treg) cells was analyzed by flow cytometry in cell suspensions obtained from the **(A, C)** spleen and **(B, D)** pancreatic lymph nodes (PLN) from adipose tissue-derived mesenchymal stem cell (ADMSC)-treated and phosphate-buffered saline (PBS)-treated mice. Cells were stained for surface markers CD4 and CD25 and subsequently for the transcription factor Foxp3. Bars represent average ± standard error of the mean. Intrasplenic route: Control-PBS, *n* = 5; R-ADMSCs, *n* = 7; NR-ADMSCs, *n* = 3. Intrapancreatic route: Control-PBS, *n* = 5; R-ADMSCs, *n* = 5; NR-ADMSCs, *n* = 7. NR-ADMSCs, nonresponder ADMSC-treated mice; R-ADMSCs, responder ADMSC-treated mice.

Treg cells have been shown to play a crucial role in regulating autoimmunity. Studies have demonstrated the role of MSCs on *in vivo/in vitro* induction and proliferation of Treg cells [28-30]. To investigate the hypothesis that the therapeutic effect of ADMSC transplantation could be associated with the expansion of Treg cells, we analyzed the frequency of CD4^+^CD25^+^Foxp3^+^ T cells in the spleen and PLN of ADMSC/PBS-treated diabetic mice. Both i.sp. and i.pc. ADMSC administration did not modulate Treg cell frequency. The percentage of CD4^+^CD25^+^Foxp3^+^ cells was similar in the spleen and PLN of R-ADMSCs, NR-ADMSCs and Control-PBS mice 70 days after the i.sp./i.pc. ADMSC injection (Figure 5).

### Intrasplenic administration of adipose tissue-derived MSCs increases TGF-β levels in pancreatic tissue of diabetic treated mice

**Figure 6:**
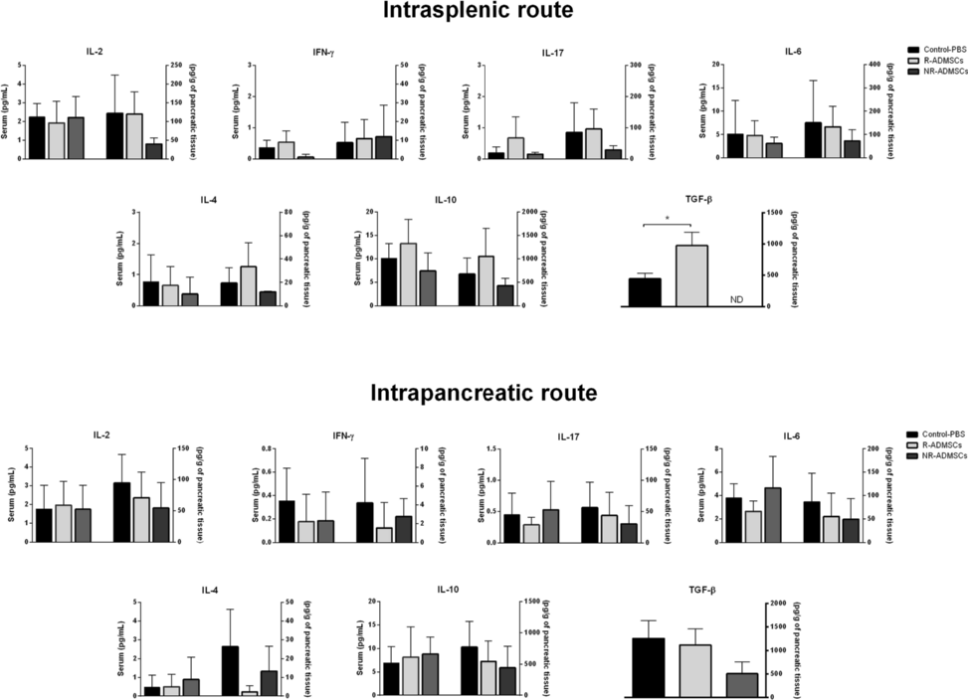
**Intrasplenic adipose tissue-derived mesenchymal stem cell administration increases transforming growth factor beta levels in pancreatic tissue of diabetic treated mice.** Blood and pancreas samples were obtained from Control and adipose tissue-derived mesenchymal stem cell (ADMSC)-treated mice 70 days after treatment. The pancreatic tissue was weighed and homogenized in the presence of proteases inhibitor. Levels of interleukin (IL)-2, interferon gamma (IFN-γ), IL-17, IL-6, IL-4 and IL-10 were measured in serum and pancreatic homogenate using the cytokine bead array (CBA) method. The transforming growth factor beta (TGF-β) level was quantified by enzyme-linked immunosorbent assay only in pancreatic tissue samples. Serum cytokine concentrations are represented by picograms of protein per milliliter (left *y* axis) and pancreatic cytokine concentrations are represented by picograms of protein per gram of pancreatic tissue (right *y* axis). Bars represent average ± standard error of the mean. Intrasplenic route: Control-PBS, *n* = 5; R-ADMSCs, *n* = 7; NR-ADMSCs, *n* = 3. Intrapancreatic route: Control-PBS, *n* = 5; R-ADMSCs*,* n = 5; NR-ADMSCs, *n* = 7. *P <0.05 (Control-PBS vs. R-ADMSCs). ND, not detected; NR-ADMSCs, nonresponder ADMSC-treated mice; PBS, phosphate-buffered saline; R-ADMSCs, responder ADMSC-treated mice.

The levels of IL-2, interferon gamma, IL-17, IL-6, IL-4 and IL-10 were unchanged in serum and pancreatic homogenate 70 days after i.sp. or i.pc. ADMSC administration. However, increased levels of TGF-β were observed in the pancreas of the R-ADMSC/i.sp. group (974.3 ± 213.3 pg/g) compared with those of the Control-PBS group (441.3 ± 86.4 pg/g; *P* = 0.02; Figure 6).

### Adipose tissue-derived MSC trafficking following injection in streptozotocin-induced diabetic mice

To allow the monitoring of ADMSC survival and biodistribution following i.sp. or i.pc. injection, we isolated ADMSCs from FVB mice constitutively expressing the bioluminescent reporter luciferase (ADMSCs^Luc+^). The obtained ADMSCs^Luc+^ displayed a typical immunophenotypic profile, differentiated towards adipogenic and osteogenic lineages, and the intensity of their bioluminescence signal was linearly proportional to the number of cells (Additional file 4). After isolation and characterization, ADMSCs^Luc+^ were administered to diabetic mice (20 days after diabetes induction) using the i.sp. or i.pc. delivery routes and the animals were subjected to BLI analysis.

**Figure 7:**
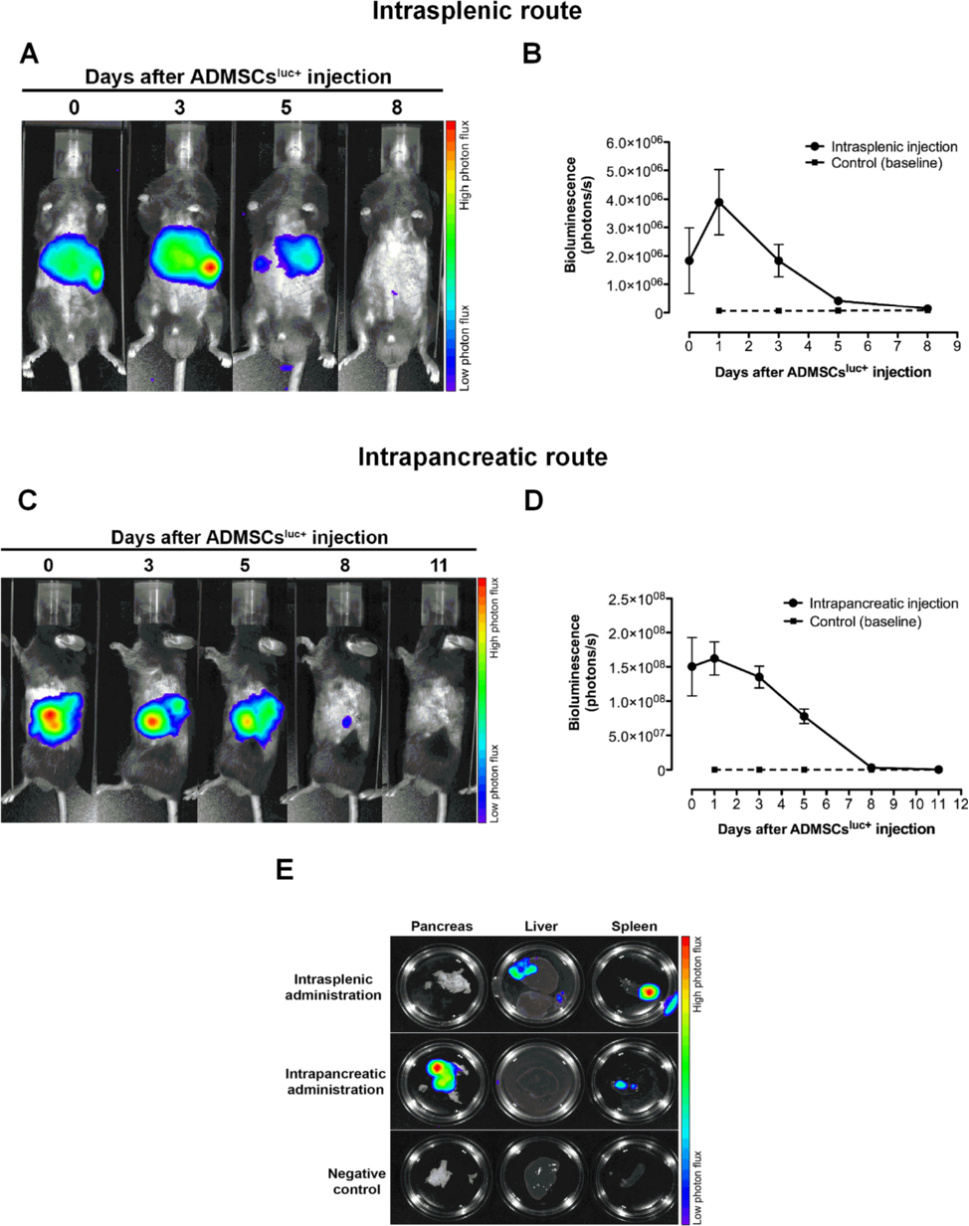
**Evaluation of** ***in vivo*** **ADMSC** ^**Luc+**^ **distribution. (A)** Bioluminescent imaging of a representative mouse following intrasplenic administration of luciferase-positive adipose tissue-derived mesenchymal stem cells (ADMSCs^Luc+^). Bioluminescent signal was detected in regions of the spleen and liver for up to 8 days. **(B)** Kinetics of ADMSC^Luc+^ survival estimated by bioluminescence quantification. The bioluminescent signal increased 24 hours after injection, followed by a continuous decrease until its complete extinction after 8 days (*n* = 5 mice). **(C)** Bioluminescent imaging of a representative mouse following intrapancreatic injection of ADMSCs^Luc+^. Bioluminescent signal was detected in the region of pancreas for up to 8 days. **(D)** Kinetics of ADMSC^Luc+^ survival estimated by bioluminescence quantification. Following injection, the number of ADMSCs^Luc+^ gradually decreased during 8 days, after which the bioluminescent signal was no longer detected (*n* = 5 mice). **(E)** *Ex vivo* bioluminescent imaging of the pancreas, liver and spleen 48 hours after ADMSC^Luc+^ injection. ADMSCs^Luc+^ injected through the splenic route occupied the spleen and liver but not the pancreas of diabetic mice. In contrast, most ADMSCs^Luc+^ injected via the intrapancreatic route remained within the pancreas, albeit relatively few cells were detected in the spleen. No ADMSCs^Luc+^ were detected in the liver after intrapancreatic injection. Negative control represents a diabetic mouse that did not receive ADMSCs^Luc+^.

Following i.sp. transplantation, ADMSCs^Luc+^ rapidly occupied the liver region while few cells were retained in the spleen, as demonstrated by *in vivo* BLI (Figure 7A). Although we observed a slight increase in bioluminescent signal 24 hours after infusion, the number of viable ADMSCs^Luc+^ continuously decreased until the eighth day, when their bioluminescence was no longer detected (Figure 7A,B). *Ex vivo* BLI of excised organs confirmed the presence of ADMSCs^Luc+^ in the spleen and liver but not in the pancreas at day 2 post ADMSC transplantation (Figure 7E, upper panel).

After i.pc. ADMSC injection, the bioluminescent signal of ADMSCs^Luc+^ was detected exclusively on the right flank of diabetic mice (Figure 7C), coinciding with the anatomical location of pancreas. Unlike the i.sp. ADMSC injection, no bioluminescent signal was detected in the liver of these mice. The i.pc.-administered ADMSCs^Luc+^ also survived during 8 days following infusion (Figure 7C,D). *Ex vivo* BLI analysis further demonstrated that most ADMSCs^Luc+^ were retained in the pancreas 48 hours after injection, albeit little bioluminescent signal was detected in the spleen (Figure 7E, middle panel). Although each delivery route resulted in distinct biodistribution patterns, ADMSCs^Luc+^ displayed similar survival kinetics *in vivo* regardless of the injection strategy.

## Discussion

In preclinical studies, the use of MSCs as a therapeutic tool to control the development and progression of several autoimmune and inflammatory diseases has been widely investigated. In rodent models of T1D, transplantation of MSCs reverted hyperglycemia, recovered pancreatic islets, increased insulin production and promoted beneficial immunologic changes [7,15-20]. Based on these promising experimental results, some clinical trials have been performed worldwide evaluating the safety and efficiency of MSCs isolated from bone marrow, umbilical cord or menstrual blood in the treatment of T1D patients [31].

One critical aspect for MSC transplantation and subsequent therapeutic efficacy is the selection of an appropriated delivery route. An optimal cell delivery technique should provide the most regenerative benefit with the lowest side effects [32]. Systemic delivery routes (intravenous/intra-arterial injections) are widely explored in experimental studies and clinical trials for several diseases. However, one problem frequently associated with these routes is the cell entrapment in the lungs as a result of mechanical and physiological aspects. Different reports showed that MSC entrapment into the mice lungs caused cessation/reduction of blood flow [25], episodes of tachypnea and apnea [24], and death in 25 to 40% of MSC-injected mice [33]. Moreover, Aguilar and colleagues showed that murine MSCs trapped within lung capillaries following systemic injection expanded and invaded into the lung parenchyma forming tumor nodules resembling osteosarcoma [34]. An enhanced MSC delivery to specific tissues is warranted and could increase the therapy efficiency, reduce the number of infused cells and consequently decrease the costs of the therapeutic product [35].

In an attempt to avoid cell entrapment into the lungs and its side effects, we tested in the present study two alternative routes of ADMSC delivery in STZ-induced diabetic mice: the i.pc. and i.sp. routes. ADMSCs were injected into diabetic mice 20 days after diabetes induction, representing a chronic phase of disease progression characterized by hyperglycemia, massive β-cell destruction and α-cell expansion with a disruption of pancreatic islet architecture [36]. After ADMSC transplantation, we evaluated the long-term therapeutic potential of allogeneic ADMSCs in the control of hyperglycemia, modulation of cytokines and Treg cells and preservation of pancreatic β-cell mass. Many of the studies that evaluated the therapeutic potential of MSCs in experimental models of diabetes only analyzed the short-term responses of cell therapy [14,16,37]. However, it is very important to evaluate the maintenance of the beneficial therapeutic responses promoted by the injected MSCs over long periods. In this sense, we followed the ADMSC-treated diabetic mice for 70 days after cell administration.

The i.pc. route was chosen in order to promote local and very precise delivery of MSCs to the injured organ. This delivery route has been used to inject hematopoietic stem cells (CD34^+^) into patients with type 2 diabetes, improving metabolic control with reduction of insulin requirements [38-40]. The therapeutic potential of intrapancreatically administered MSCs was evaluated in murine and swine models of diabetes. Multipoint pancreatic injections of porcine bone marrow-derived MSCs decreased blood glucose levels, improved blood insulin concentration and increased the number of islets in diabetic pigs on day 60 post transplantation [41]. Katuchova and colleagues transplanted syngeneic bone marrow-derived MSCs in different regions of the pancreas (head, tail or the whole pancreas) and observed short-term improvement in hyperglycemia in experimental diabetic treated rats [42].

Alternatively, MSCs injected into the spleen (i.sp. route) might be delivered to the pancreas via splenic arterial blood circulation since the tail and the body of the pancreas receive their blood supply from the splenic artery’s pancreatic branches. MSCs in the splenic microenvironment could also promote the modulation of splenocytes, leading to a decreased immune response against pancreatic β cells. Moreover, different studies suggest that the spleen can influence β-cell proliferation [43-45]. The administration of MSCs in experimental models and clinical protocols by the i.sp. route is underexplored and has been tested mainly for the treatment of liver diseases in humans and experimental models [46,47].

In this study, allogeneic ADMSCs injected by both delivery routes were able to decrease the blood glucose levels in diabetic treated mice. The i.sp. ADMSC transplantation promoted hyperglycemia reversion in 70% of diabetic mice (responders), improved the number and size of pancreatic islets and increased circulating-insulin levels. ADMSCs injected by the i.pc. route reverted hyperglycemia in 42% of diabetic mice and improved the size of pancreatic islets. However, the insulin levels were similar among the i.pc. experimental groups 70 days after cell/PBS administration. The insulin levels may vary along the day and the measurement performed in this study reflects just a snapshot of that moment. A daily profile of insulin levels would be interesting to match the results obtained for islet size and *in situ* insulin staining. Nonresponder treated mice remained hyperglycemic during the follow-up and the factors that make them unresponsive to ADMSC action were not investigated in this study. The observation of diabetic rodents refractory to MSC treatment was also reported by other groups; however, the mechanisms involved in this phenomenon are still unclear [7,20].

In our study, mice responsive to treatment showed a significant decrease in blood glucose levels 6 days after i.sp. or i.pc. ADMSC administration and remained long-term normoglycemic. This sudden drop in blood glucose levels observed a few days after MSC infusion has been reported by other studies. Ezquer and colleagues observed normoglycemic levels in STZ-induced diabetic mice 7 days after the intravenous administration of 0.5 × 10^6^ MSCs [15]. Similarly, the administration of two doses of 2 × 10^6^ human MSCs into the left ventricle of STZ-induced nonobese diabetic (NOD)/SCID diabetic mice reverted hyperglycemia 7 days after the second MSC infusion [14]. NOD mice treated with MSCs by the intraperitoneal or intravenous route exhibited significant decrease in blood glucose levels 7 days after cell therapy [7,18]. MSCs thus appear to exert their therapeutic function rapidly after administration in diabetic mice.

The i.sp. or i.pc. injection of ADMSCs promoted an improvement in glycemic control in R-ADMSCs; however, these animals do not reach euglycemic levels like those presented by nondiabetic mice (wild type). Our ADMSCs therapy decreased blood glucose levels, improved β-cell mass and insulin production but did not reverse the diabetes completely. This incomplete reversion of diabetes promoted by MSC therapy was reported by other researchers [15,37,48] and may be a result of an incomplete pancreatic islet regeneration, and a second dose of MSCs would represent a good strategy to improve therapy efficiency [15].

MSCs have the ability to induce/expand the population of Treg cells, both *in vitro* and *in vivo* [28-30]. Treg cells play a fundamental role in immunological homeostasis by suppressing the response against self-antigens and limiting excessive immune reaction. Problems in ontogenesis or function of Treg cells result in development of autoimmune and inflammatory diseases in humans and animal models [49]. In the present study, regardless of the route of ADMSC administration, no late alterations in the frequency of Treg cells were observed in the spleen and PLN of diabetic treated mice. The concentration of serum and pancreatic cytokines also remained unchanged after ADMSC transplantation. Contrasting with these observations, increased frequency of CD4^+^ Foxp3^+^ cells [16] and high IL-10 levels [18] were previously reported in MSC-treated NOD mice a few days (5 to 7 days) after MSC transplantation. However, no changes in Treg cell frequency were observed when the analyses were performed 14 or 28 days after the administration of MSCs [17,18]. Our analyses were performed 70 days after ADMSC administration, which may represent a period of time too long to detect alterations in Treg cell frequency and in proinflammatory cytokines levels. The inflammatory process (insulitis) and changes in different T-cell subsets occur during the initial phases of disease development in the murine model of diabetes induced by STZ [36] and further experiments should be performed early after cell transplantation to characterize immediate immune alterations promoted by injected MSCs.

The administration of ADMSCs by i.sp. route modulated the levels of TGF-β in the pancreatic tissue of responder treated mice. TGF-β is a regulatory cytokine that plays pleiotropic roles in immune system [50] and promotes protection against autoimmune diabetes [51]. In a STZ-induced experimental diabetes model, increased levels of TGF-β could decrease the inflammatory process in the pancreatic islets, thereby allowing pancreatic recovery.

According to our data, i.sp. transplantation of ADMSCs was able to promote good therapeutic results even remaining for a short time (8 days) in the recipients. The observation of diabetes reversion seems to be the result of ADMSCs paracrine actions rather than ADMSCs transdifferentiation since no luciferase-positive cells were found in the pancreas of diabetic treated mice after i.sp. ADMSC injection. This finding is in accordance with data obtained by other research groups reporting no evidence of *in vivo* transdifferentiation of injected MSCs into pancreatic β cells in experimental models of diabetes [15,19,21,52]. The exact mechanism by which ADMSCs induced hyperglycemia reversion in our model is not yet clear. The expression of anti-inflammatory [7], antiapoptotic, proangiogenic [20] and mitogenic [19] molecules by transplanted MSCs might represent mechanisms that induce and improve pancreatic repair in a diabetes setting [12]. The beneficial effects provided by MSC transplantation can thus be maintained for extended periods of time, with no need for MSC survival and maintenance in the injury site.

Surprisingly, we observed a twofold increase in the bioluminescent signal 24 hours after i.sp. ADMSC^Luc+^ transplantation. Similarly, Nakabayashi and colleagues reported a rapid increase in bioluminescence 12 hours after MSC transplantation in a mouse model of muscular injury [53]. Since the doubling time of ADMSCs^Luc+^ is higher than 24 hours (data not shown), it is unlikely that this initial increase of bioluminescent signal is due to ADMSC^Luc+^ proliferation *in vivo*. Instead, we speculate that the vascular entrapment of intrasplenically injected ADMSCs^Luc+^ might have reduced the blood perfusion in the liver and spleen, thereby reducing the amount of luciferin reaching the injected cells at day 0. With ADMSC extravasation to parenchyma, which occurs within hours [54], the diffusion of luciferin might have been reestablished, leading to increased bioluminescence 24 hours after injection. Alternatively, it has been proposed that this initial rapid increase in bioluminescence might be a consequence of neovascularization driven by injected MSCs, enhancing the amount of luciferin that reaches the implanted cells [53].

Following i.sp. ADMSC transplantation, cells were retained in the spleen and liver of diabetic treated mice. It has been shown by different studies that these organs can influence β-cell proliferation. A role for the spleen in β-cell regeneration was suggested by clinical data showing that the incidence of diabetes was significantly higher in patients undergoing partial pancreatectomy and splenectomy than in those undergoing pancreatectomy alone. The authors thus suggest that splenic preservation might delay the onset of diabetes [43]. Kodama and colleagues reported that spleen resident stem cells were capable of differentiating into β cells, restoring β-cell mass and reverting diabetes in NOD mice [44]. The effect of the spleen in the restoration of pancreatic β-cell function was investigated in severely diabetic adult C57BL/6 mice by Yin and colleagues. When syngeneic islets were transplanted into these diabetic mice under a single kidney capsule, a stable restoration of euglycemia was observed associated with increased β-cell mass, as well as β-cell hypertrophy and proliferation. Importantly, the restoration of islet cell function was facilitated by the presence of the spleen; however, it was not due to the direct differentiation of spleen-derived cells into β cells [45]. Recently, Yi and colleagues identified the hormone betatrophin that is primarily expressed in the liver and fat. Transient expression of betatrophin in mouse liver significantly and specifically promoted pancreatic β-cell proliferation, expanded β-cell mass and improved glucose tolerance in insulin-resistant diabetic mice [55]. The retention of ADMSCs in the spleen and liver could contribute to the indirect effects that these organs play in the expansion of pancreatic β cells. The production of bioactive molecules by ADMSCs could induce the production of growth factors by hepatocytes/splenocytes to act in a paracrine fashion, stimulating endogenous progenitors to differentiate into insulin-producing β cells.

The administration of MSCs by the i.pc. route was not as efficient as the i.sp. route in promoting diabetes reversion, suggesting that MSCs do not need to be retained in the damaged pancreas to exert their therapeutic function. Using a model of myocardial infarction and spontaneous diabetes, Lee and colleagues showed that intravenously injected human MSCs were trapped in the lungs and were activated to secrete the anti-inflammatory molecule tumor necrosis factor alpha-induced protein TSG-6 at this site. TSG-6 produced by MSCs retained in the lungs improved myocardial function and delayed the onset of diabetes, decreased insulitis and suppressed T-helper type 1 cell polarization in MSC-treated mice [56,57]. In this sense, we can suggest that administered MSCs do not need to be retained at the site of injury, but should be efficiently stimulated, even distant, to produce trophic factors that will act on other cells in the body, promoting tissue repair.

## Conclusions

ADMSCs injected by the i.sp. or i.pc. delivery routes were able to decrease blood glucose levels and improve glucose tolerance in STZ diabetic mice. The i.sp. ADMSC administration attenuated the hyperglycemia in 70% of diabetic treated mice and stimulated insulin production by pancreatic β cells. The Treg cell population remained unchanged after ADMSC administration but pancreatic TGF-β levels were increased in i.sp. ADMSC-treated mice. ADMSCs injected by these delivery routes remained a few days in the recipient and none of them were detected in the pancreas for long periods. Considering the potential role of MSCs in the treatment of several disorders, this study reports alternative delivery routes that circumvent cell entrapment into the lungs, promoting beneficial therapeutic responses in ADMSC-treated diabetic mice. The i.sp. route for stem cell delivery should be further explored in the diabetes setting and may represent a promising therapeutic approach for patients with T1D in the future.

## Additional files

Additional file 1: Figure S1. Showing the experimental design. Diabetes was induced in C57BL/6 male mice after 5 consecutive daily injections of STZ. Twenty days after diabetes induction, diabetic mice were treated with 1 × 10^6^ ADMSCs injected by intrasplenic (*n* = 10) or intrapancreatic (*n* = 12) delivery routes. Control groups of diabetic mice were injected with PBS by intrasplenic (*n* = 5) or intrapancreatic (*n* = 5) delivery routes. Nonfasting blood glucose levels were frequently determined. Seventy days after PBS/ADMSC administratio,n mice were sacrificed, different tissue samples were collected and analyzed and the distribution of ADMSCs along organs was observed. Additional file 2: Figure S2. Showing the characterization of ADMSCs. (A) Morphology of *in vitro*-expanded MSCs isolated from adipose tissue (ADMSCs) of Balb/c male mice; magnification 200×. (B) Representative immunophenotypic profile of ADMSCs at the fourth passage. *In vitro* (C) adipocyte (Sudan II-Scarlet staining) and (D) osteocyte (Von Kossa staining) differentiation; magnification 200 × . Additional file 3: Figure S3. Showing Ki-67 expression in pancreatic cells. Pancreata from the Control group and R-ADMSCs were collected 70 days after the treatment. Pancreatic tissue sections were analyzed by immunohistochemistry with anti-Ki-67 antibody to evaluate proliferating pancreatic islet cells. Representative images of pancreatic islets from the Control-PBS group (left), the R-ADMSCs group by the intrasplenic route/i.sp. (middle) and the R-ADMSCs group by the intrapancreatic route/i.pc. (right) are shown. Magnification 200 × . Additional file 4: Figure S4. Showing the characterization of ADMSCs^Luc+^. (A) Morphology of *in vitro* expanded MSCs isolated from adipose tissue of FVB^Luc+^ mice (ADMSCs^Luc+^); magnification 200×. (B) Representative immunophenotypic profile of ADMSCs^Luc+^ at the fourth passage. *In vitro* (C) adipocyte and (D) osteocyte diferentiation, original magnification 200×. (E) *In vitro* bioluminescent imaging demonstrating that ADMSCs^Luc+^ expressed biologically active luciferase. (F) Linear regression between the number of ADMSCs^Luc+^ and their bioluminescent signal. The bioluminescence was directly and linearly proportional to the number of ADMSCs^Luc+^ (linear regression test, *R* ^2^ = 0.99, *P* <0.0001).

## Abbreviations

ADMSC: adipose tissue-derived mesenchymal stem cell
AUC: area under the curve
BLI: bioluminescent imaging
FBS: fetal bovine serum
GTT: glucose tolerance test
IL: interleukin
i.pc.: intrapancreatic
i.sp.: intrasplenic
Luc^+^: luciferase positive
MSC: mesenchymal stem cell
NOD: nonobese diabetic
NR-ADMSCs: nonresponder adipose tissue-derived mesenchymal stem cell-treated mice
PBS: phosphate-buffered saline
PLN: pancreatic lymph nodes
R-ADMSCs: responder adipose tissue-derived mesenchymal stem cell-treated mice
STZ: streptozotocin
T1D: type 1 diabetes mellitus
TGF-β: transforming growth factor beta
Treg: regulatory T
