# Supplementary figures and images for: Therapeutic efficacy and biodistribution of allogeneic mesenchymal stem cells delivered by intrasplenic and intrapancreatic routes in streptozotocin-induced diabetic mice
Source: Stem Cell Res Ther. 2015 Mar 14;6(1):31. doi: 10.1186/s13287-015-0017-1 (PMC4432770; doi:10.1186/s13287-015-0017-1)

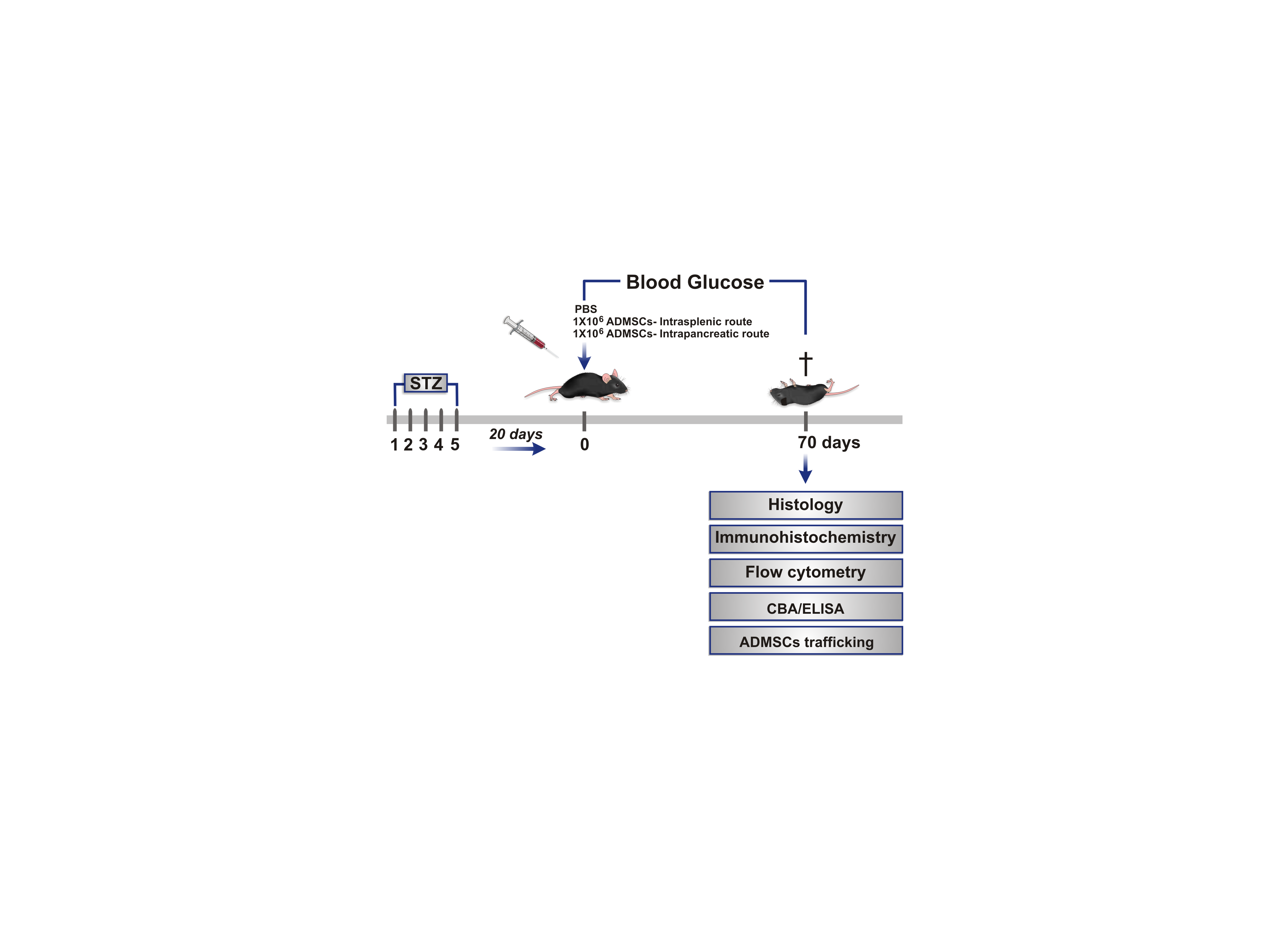

Supplement: Additional file 1: Figure S1. — Showing the experimental design. Diabetes was induced in C57BL/6 male mice after 5 consecutive daily injections of STZ. Twenty days after diabetes induction, diabetic mice were treated with 1 × 10^6^ ADMSCs injected by intrasplenic (n = 10) or intrapancreatic (n = 12) delivery routes. Control groups of diabetic mice were injected with PBS by intrasplenic (n = 5) or intrapancreatic (n = 5) delivery routes. Nonfasting blood glucose levels were frequently determined. Seventy days after PBS/ADMSC administratio,n mice were sacrificed, different tissue samples were collected and analyzed and the distribution of ADMSCs along organs was observed. [file 13287_2015_17_MOESM1_ESM.tiff]

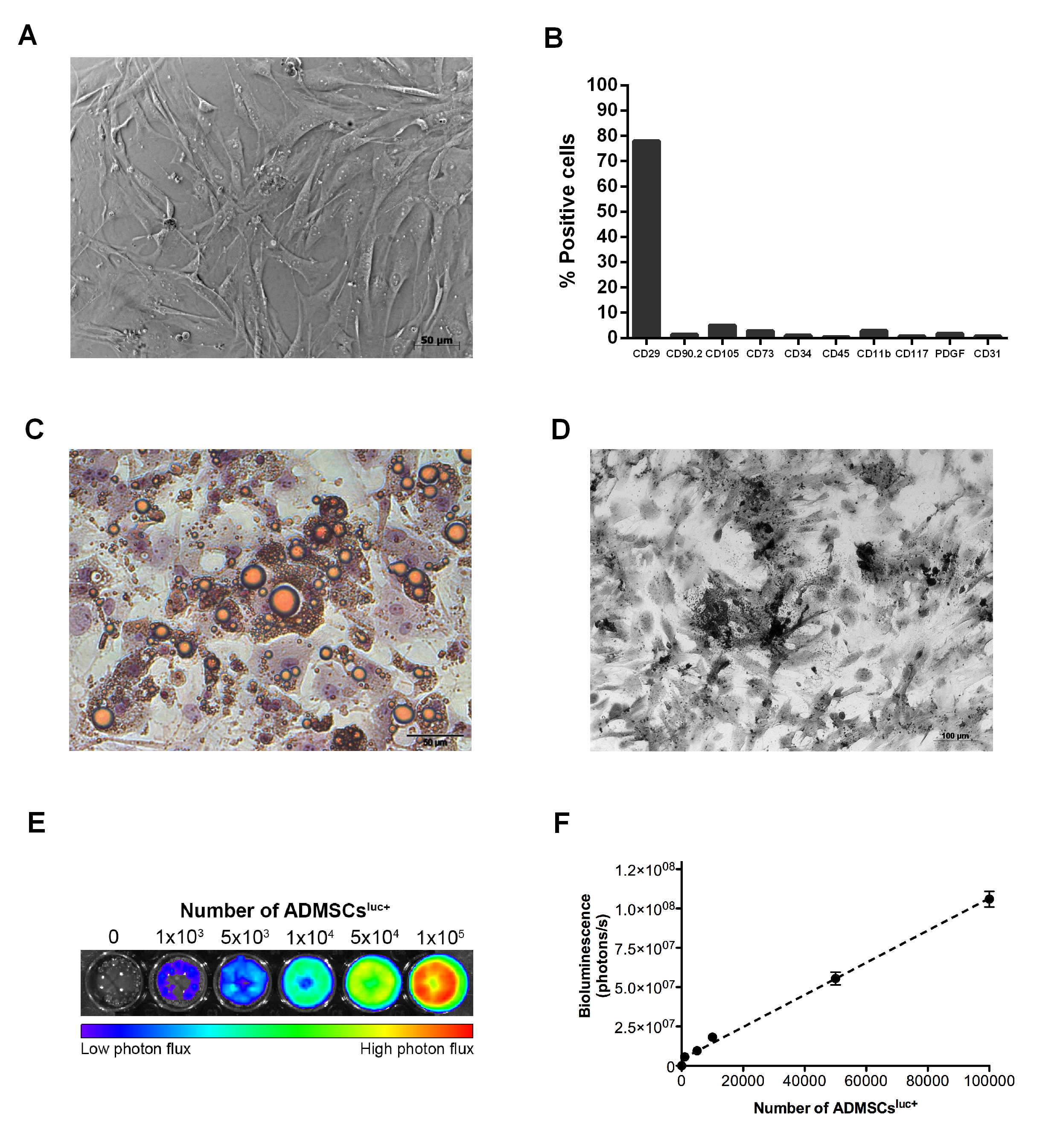

Supplement: Additional file 4: Figure S4. — Showing the characterization of ADMSCsLuc+. (A) Morphology of in vitro expanded MSCs isolated from adipose tissue of FVBLuc+ mice (ADMSCsLuc+); magnification 200×. (B) Representative immunophenotypic profile of ADMSCsLuc+ at the fourth passage. In vitro (C) adipocyte and (D) osteocyte diferentiation, original magnification 200×. (E) In vitro bioluminescent imaging demonstrating that ADMSCsLuc+ expressed biologically active luciferase. (F) Linear regression between the number of ADMSCsLuc+ and their bioluminescent signal. The bioluminescence was directly and linearly proportional to the number of ADMSCsLuc+ (linear regression test, R 2 = 0.99, P <0.0001). [file 13287_2015_17_MOESM4_ESM.jpeg]
